# Joint Beamforming and Trajectory Optimization Algorithm for RSMA-UAV-Enabled Integrated Sensing and Communication System

**DOI:** 10.3390/s26165075

**Published:** 2026-08-10

**Authors:** Shun Wang, Qi Zhu

**Affiliations:** 1 Jiangsu Key Laboratory of Wireless Communications, Nanjing University of Posts and Telecommunications, Nanjing 210003, China; 1224014411@njupt.edu.cn; 2 Engineering Research Center of Health Service System Based on Ubiquitous Wireless Networks, Nanjing University of Posts and Telecommunications, Nanjing 210003, China

**Keywords:** integrated sensing and communication, beamforming, rate-splitting multiple access, UAV trajectory, successive convex approximation

## Abstract

Integrated sensing and communication (ISAC) enables concurrent wireless communication and target sensing using shared hardware and spectrum resources, and is regarded as a key technology for next-generation mobile networks. To address the challenge of coordinating sensing and communication in multiuser scenarios, this paper investigates an unmanned aerial vehicle (UAV)-enabled ISAC system employing rate-splitting multiple access (RSMA) and proposes a joint beamforming and trajectory optimization framework. Specifically, ground users are first clustered subject to a capacity–diameter constraint, and the UAV employs RSMA to serve users within each cluster. The sensing performance is characterized by the composite transmit beampattern gain in the target direction contributed by the common stream and all private streams. Under constraints on users’ downlink rates, transmit power, and sensing quality, we formulate an optimization problem to maximize the average downlink rate. By adopting a block coordinate descent (BCD) framework, the resulting non-convex problem is decomposed into three subproblems, namely cluster scheduling, rate allocation and beamforming, and UAV trajectory optimization. These subproblems are then solved via linear programming, semidefinite relaxation, and successive convex approximation, respectively. Numerical results demonstrate that, while satisfying the sensing-quality requirement, the proposed algorithm significantly improves the achievable system downlink rate.

## 1. Introduction

In recent years, with the emergence of new intelligent applications such as autonomous driving, unmanned aerial vehicles communications, intelligent transportation, and telemedicine, communication networks have faced an increasing demand for the joint capability of environmental sensing and information transmission [1]. Conventional communication systems and radar sensing systems are typically deployed independently, which leads to redundant occupation of spectrum and hardware resources and also limits the energy efficiency and real-time performance of the overall system [2]. To address these issues, integrated sensing and communication (ISAC) has emerged as a promising paradigm [3], which enables the co-design of communication and sensing on a shared spectrum and hardware platform. By sharing waveforms, signal processing, and resource scheduling, ISAC can improve spectrum efficiency, energy efficiency, and hardware utilization, and further achieve synergistic gains through communication-assisted sensing and sensing-aided communication. Therefore, ISAC is widely regarded as one of the key enabling technologies for next-generation mobile communication systems [4].

Beamforming plays a crucial role in improving the performance of ISAC systems. Reference [5] provides a comprehensive survey of the fundamental limits of ISAC, including the sensing and communication performance metrics, theoretical bounds, and the fundamental tradeoffs between the two functionalities. Reference [6] considers a shared array multiuser multiple input multiple output (MIMO) communication and MIMO radar scenario, and minimizes the radar beampattern error while satisfying the signal to interference plus noise ratio (SINR) requirements of all communication users. In [7], for a downlink ISAC system, a multiobjective beamforming formulation is established with the weighted beampattern gain and the communication rate as objectives; by leveraging semidefinite relaxation (SDR) and generalized eigenvalue decomposition, a structured optimal beamforming solution is obtained. Reference [8] extends the problem to a reconfigurable intelligent surface (RIS)-aided dual-functional radar communication scenario, where the transmit waveform, receive filter, and RIS phase shifts are jointly optimized under constraints on the transmit power and RIS elements. Toward massive arrays and more complex scenarios, ref. [9] studies near-field ISAC under a spherical wave channel model, and designs near-field beamforming via successive convex approximation by minimizing the mismatch between the desired and the actual two dimensional range–angle beampattern. From a multiobjective optimization perspective, ref. [10] further formulates a Pareto optimization problem based on the multiuser communication SINR and an upper bound on the sensing mutual information for multitarget sensing, and obtains optimal beamforming solutions under different weights via SDR and max–min utility optimization. In an uplink MIMO-ISAC scenario, ref. [11] addresses the coexistence of communication signals and radar echoes, and proposes an interference cancelation-based detection estimation scheme as well as a minimum mean square error-based joint receiver design.

Unmanned aerial vehicles (UAVs) offer advantages such as line-of-sight coverage, flexible mobility, and rapid deployment, thereby providing ISAC with an adjustable spatial dimension and a high degree of freedom; as a result, UAV-enabled ISAC has attracted increasing research interest. A recent overview of the opportunities, architectures, and design challenges of UAV-enabled ISAC is provided in [12]. Reference [13] extends beamforming design to a UAV empowered adaptable ISAC system by introducing adaptive mechanisms for the maneuvering trajectory and sensing duration, and jointly optimizes beamforming and trajectory under power and sensing threshold constraints. Reference [14] considers two operation modes, namely quasi-static placement and fully dynamic flight, and jointly optimizes the trajectory and beamforming to maximize the weighted communication rate subject to sensing beampattern gain and flight constraints. Reference [15] investigates a UAV cooperative architecture integrating sensing, wireless power transfer, and communications, where environmental sensing, wireless energy provisioning, and uplink data offloading are accomplished via a two-stage protocol. References [16,17] focus on multi-UAV cooperation and periodic ISAC tasks, and jointly design UAV trajectories, user association, bandwidth and power allocation, and sensing scheduling, demonstrating the potential of balancing communication and sensing at the task level through spatial mobility and time-domain scheduling.

In scenarios with concurrent multiuser access and parallel sensing tasks, it is difficult to further mitigate co-channel interference by relying solely on spatial beamforming and time-slot scheduling. Rate-splitting multiple access (RSMA) provides a flexible interference-management framework by partially decoding interference and partially treating it as noise [18,19], which makes it suitable for multiuser ISAC systems. In a cellular ISAC setting, ref. [20] incorporates RSMA and jointly optimizes the waveform and precoding via SDR, successive convex approximation (SCA), and related techniques; however, it is restricted to a fixed base station. Reference [21] considers a single UAV RSMA-ISAC system and jointly optimizes the UAV deployment location and transmit beamforming to maximize the energy efficiency under sensing accuracy constraints. Reference [22] studies a multi-UAV cooperative RSMA-ISAC scenario and jointly designs user association, UAV placement, beamforming, and rate allocation via convex optimization. However, refs. [21,22] optimize only the UAV positions rather than the flight trajectories. Reference [23] considers a mobile RSMA-UAV-ISAC system and jointly optimizes scheduling, power allocation, and UAV trajectory; however, sensing and communication are performed in a time-division manner, and the common and private beamforming vectors are not jointly designed. To further clarify the differences between the present work and the closely related RSMA-UAV-ISAC studies, a comparison is provided in Table 1. In this paper, we consider a UAV-ISAC scenario where the communication users simultaneously serve as sensing targets, and the transmit beampattern gain in the target direction is adopted as the sensing metric. We propose an RSMA-based joint optimization framework for user scheduling, beamforming, common-rate allocation, and UAV trajectory to improve the system communication rate. The main contributions of this work are summarized as follows:

(1) We formulate a clustered RSMA-based joint design framework for a mobile UAV-enabled integrated sensing and communication system, where ground users simultaneously serve as communication terminals and sensing targets. Unlike existing static-deployment or time-division UAV-ISAC schemes, the proposed framework jointly incorporates capacity–diameter-constrained user clustering, time-slot-based cluster scheduling, RSMA common-rate allocation, common and private beamforming, and continuous UAV trajectory design. Moreover, the common and private streams are jointly exploited to form the sensing beampattern, enabling simultaneous communication and sensing using the same transmitted waveform.
(2) We develop a block-wise alternating optimization algorithm for the formulated mixed-integer non-convex problem by exploiting the specific structures of different variable blocks. The problem is decomposed into three subproblems, namely cluster scheduling, RSMA rate allocation and beamforming, and UAV trajectory optimization. The scheduling subproblem is handled through continuous relaxation and linear programming. The rate allocation and beamforming subproblem is solved by combining semidefinite relaxation, successive convex approximation, and a rank-penalty strategy, while the UAV trajectory subproblem is addressed using successive convex approximation.
(3) By alternately updating the three variable blocks, we obtain a converged feasible solution for cluster scheduling, common-rate allocation, common and private beamforming, and UAV trajectory. Simulation results demonstrate that the proposed joint design effectively improves the average system communication rate while satisfying the sensing-quality requirement. Under the considered simulation settings, the proposed algorithm achieves an average communication-rate improvement of approximately 8% compared with [23].

The remainder of this paper is organized as follows. Section 2 presents the clustered RSMA-UAV-ISAC scenario and the signal models for communication and sensing. Section 3 develops the BCD-based joint optimization algorithm for user scheduling, common-rate allocation, common and private beamforming, and UAV trajectory. Section 4 provides simulation results and discussions. Finally, Section 5 concludes the paper.

Notations: In this paper, the lowercase bold letter a and the uppercase bold letter A represent vectors and matrices, respectively. $A^{*}$, $A^{T}$ and $A^{H}$ denote the conjugate, the transpose, and the conjugate transpose of A, respectively. rank(A) and tr(A) represent the rank and trace of A, respectively. A≥0 indicates that A is a positive semidefinite Hermitian matrix. $n\sim CN\left(\mu ,\sigma^{2}\right)$ denotes a random vector that follows a complex Gaussian distribution with mean μ and covariance $\sigma^{2}$. The statistical expectation is denoted as E(⋅). $||\cdot |{|}_{F}$ denotes the Frobenius norm, and A⊗B is the Kronecker product between A and B.

## 2. System Model

**Figure 1:**
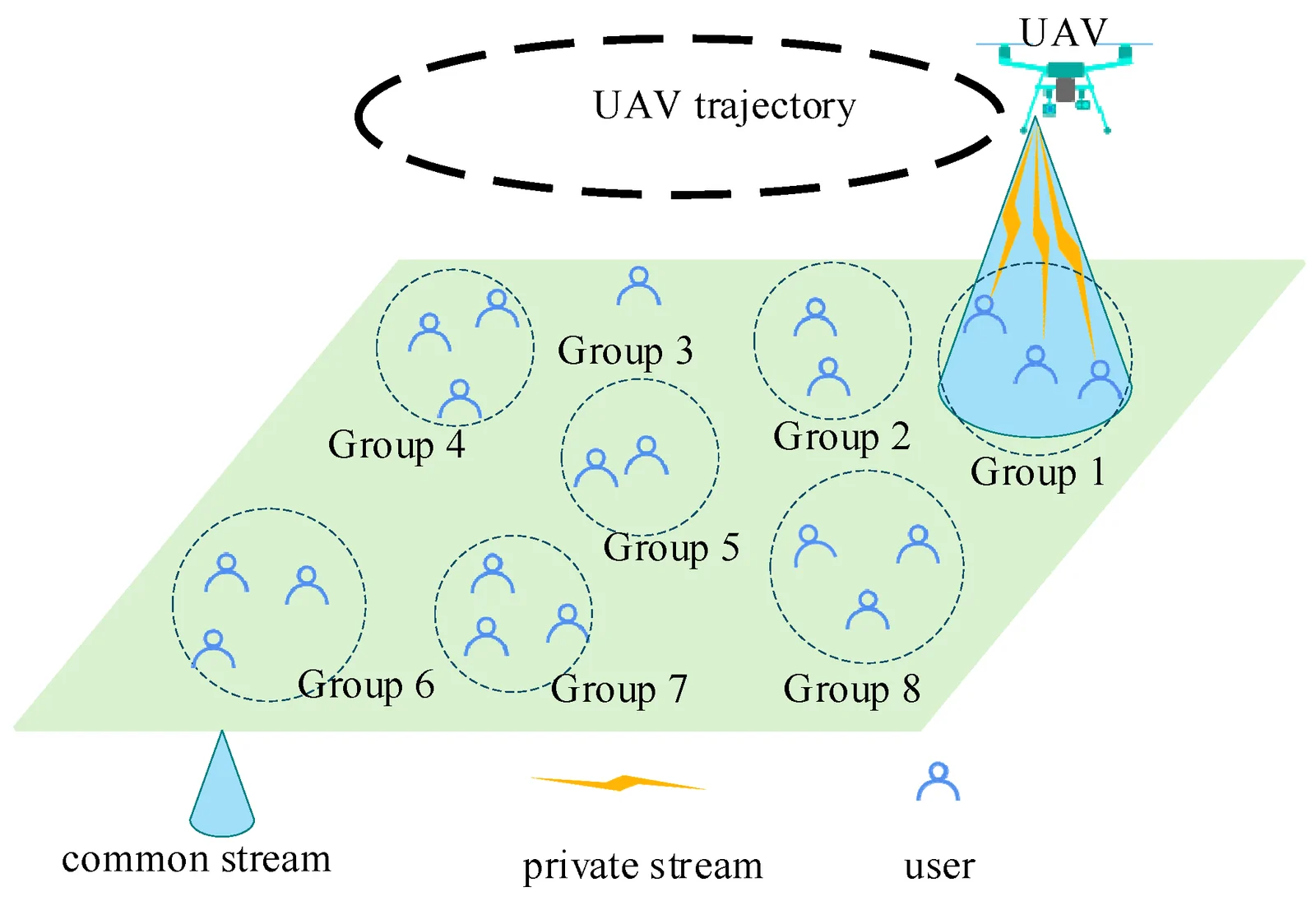
RSMA-assisted UAV-ISAC system.

As shown in Figure 1, we consider a UAV-assisted integrated sensing and communication system consisting of one unmanned aerial vehicle and multiple ground users. In this system, K single antenna users are randomly distributed, and the user set is κ={1,…,K}. The users are partitioned into L clusters, where the number of users in each cluster does not exceed m. The cluster set is ℜ={1,…L}, and the number of users in cluster l is $m_{l}$, with $m_{l}\leq m,\;\forall l\in ℜ$. Let $u_{l,k}={\left[x_{l,k},y_{l,k},0\right]}^{T}$ denote the three dimensional position vector of user k in cluster l, whose horizontal location is ${\left[x_{l,k},y_{l,k}\right]}^{T}$. The UAV flies at a fixed altitude H over a flight period T, which is equally divided into N time-slots of length $\delta_{t}=T/N$. The time slot index is n∈{1,2,…,N}. Since each time slot is sufficiently short, the UAV is assumed to remain quasi-static within each slot. The horizontal position of the UAV at time slot n is denoted by $q_{U}[n]=(x_{U}[n],y_{U}[n])$, where n∈{1,2,…,N}. The UAV is equipped with a uniform planar array (UPA) with a total of $M=M_{x}\times M_{y}$ antennas, where $M_{x}$ and $M_{y}$ are the numbers of antenna elements along the x and y axes, respectively. The inter element spacing is λ/2, where λ is the carrier wavelength. The UPA plane is parallel to the ground, and the main lobe points vertically downward; the UAV attitude is assumed unchanged within each time slot [21]. The UAV is assumed to start and end at the same location, i.e., $q_{U}[1]=q_{U}[N]=q_{0}$. The UAV motion between two consecutive time slots satisfies $||q_{U}[n]-q_{U}[n-1]||\leq v_{\max}\delta_{t}$, where $v_{\max}$ is the maximum UAV speed. In each time slot n, the UAV employs rate splitting multiple access for downlink communication with the users in the scheduled cluster, and uses the same transmitted signal to sense these in-cluster users.

### 2.1. Communication Model

We assume that the downlink communication link between the UAV and the ground users is dominated by the line of sight component, and thus the air to ground channel follows the free space path loss model. In time slot n, the large scale path loss from the UAV to user k in cluster l is given by

(1) $$\beta_{l,k}[n]=\beta_{0}{d_{l,k}}^{-2}[n]$$

where $\beta_{0}$ denotes the path loss constant at the reference distance of 1 m, and $d_{l,k}[n]$ denotes the distance between the UAV and this user in time slot n, which is expressed as

(2) $$d_{l,k}[n]=\sqrt{{\left(x_{U}[n]-x_{l,k}\right)}^{2}+{\left(y_{U}[n]-y_{l,k}\right)}^{2}+H^{2}}$$

Let the azimuth and elevation angles between the UAV and user k in cluster l at time slot n be denoted by $\phi_{l,k}[n]$ and $\theta_{l,k}[n]$, respectively. Their direction cosines are uniquely determined by $\phi_{l,k}[n]$ and $\theta_{l,k}[n]$ as

(3) $$\begin{array}{l} \sin\theta_{l,k}[n]\cos\phi_{l,k}[n]=\frac{x_{U}[n]-x_{l,k}}{d_{l,k}[n]}\\ \sin\theta_{l,k}[n]\sin\phi_{l,k}[n]=\frac{y_{U}[n]-y_{l,k}}{d_{l,k}[n]} \end{array}$$

The transmit array response vector of the UAV toward user k in cluster l at time slot n is given by [17]

(4) $$\begin{array}{ll} a^{H}\left(q_{U}[n],u_{l,k}\right) & ={\left[1,e^{-j\pi \sin\theta \cos\phi },\ldots ,e^{-j\pi (M_{x}-1)\sin\theta \cos\phi }\right]}^{T}\\ & ⊗{\left[1,e^{-j\pi \sin\theta \sin\phi },\ldots ,e^{-j\pi (M_{y}-1)\sin\theta \sin\phi }\right]}^{T} \end{array}$$

where $\theta =\theta_{l,k}[n]$ and $\phi =\phi_{l,k}[n]$.

Accordingly, the channel gain between the UAV and user k in cluster l at time slot n can be expressed as

(5) $${h_{l,k}}^{H}[n]=\sqrt{\beta_{l,k}[n]}a^{H}\left(q_{U}[n],u_{l,k}\right)$$

Let $\alpha_{l}[n]\in \{0,1\}$ indicate whether cluster l is scheduled at time slot n if the UAV communicates with the users in cluster l during slot n, then $\alpha_{l}[n]=1$; otherwise, $\alpha_{l}[n]=0$. Since the UAV communicates with at most one cluster in each time slot, we have

(6) $$\sum_{l=1}^{L}\alpha_{l}[n]\leq 1,n=1,\ldots ,N$$

In each time slot, the UAV employs a one-layer RSMA scheme for downlink transmission to the users in the scheduled cluster. For each user $u_{l,k}$, its message $w_{l,k}$ is split into a common part and a private part. The common parts of all users are jointly encoded into one common data stream $s_{c,l}[n]$, while the private part of each user is separately encoded into a private data stream $s_{p,l,k}[n]$. Hence, the transmit signal of the UAV at time slot n can be written as

(7) $$x[n]=\sum_{l=1}^{L}\alpha_{l}[n](w_{c,l}[n]s_{c,l}[n]+\sum_{k=1}^{m_{l}}w_{p,l,k}[n]s_{p,l,k}[n])$$

Here, $w_{c,l}[n]$ denotes the common stream beamforming vector for cluster l, and $w_{p,l,k}[n]$ denotes the private stream beamforming vector for user k in cluster l. All information symbols are independently distributed and follow circularly symmetric complex Gaussian distributions with zero mean and unit power.

If the maximum transmit power of the UAV is $P_{\max}$, then

(8) $$\sum_{l=1}^{L}\alpha_{l}[n](||w_{c,l}[n]|{|}^{2}+\sum_{k=1}^{m_{l}}||w_{p,l,k}[n]|{|}^{2})\leq P_{\max},\forall n$$

Accordingly, the received signal at user k in cluster l at time slot n is

(9) $$y_{l,k}[n]=h_{l,k}^{H}[n]x[n]+n_{l,k}[n]$$

where $n_{l,k}[n]\sim CN\left(0,\sigma^{2}\right)$ denotes the additive white Gaussian noise (AWGN) at the receiver.

When $\alpha_{l}[n]=1$, the users in cluster l first decode the common stream by treating all private streams as interference. In this case, the signal to interference plus noise ratio of the common stream at user $u_{l,k}$ can be expressed as

(10) $$\gamma_{l,k}^{c}[n]=\frac{|h_{l,k}^{H}[n]w_{c,l}[n]{|}^{2}}{\sum_{i=1}^{m_{l}}|h_{l,k}^{H}[n]w_{p,l,i}[n]{|}^{2}+\sigma_{l,k}^{2}}$$

Thus, the achievable rate of the common stream at user $u_{l,k}$ is

(11) $$R_{l,k}^{c}[n]=\log_{2}(1+\gamma_{l,k}^{c}[n])$$

To ensure that all users in cluster l can successfully decode the common stream, the common stream achievable rate of cluster l is taken as the minimum among the in-cluster users, which can be written as

(12) $$R_{l}^{com}[n]=\underset{k\in K_{l}}{\min}R_{l,k}^{c}[n]$$

Let $c_{l,k}[n]\geq 0$ denote the common information rate allocated to user $u_{l,k}$ in cluster l at time slot n. Then we have

(13) $$\sum_{k=1}^{m_{l}}c_{l,k}[n]\leq R_{l}^{com}[n],\forall l,n$$

After successfully decoding and removing the common stream, the SINR of the private stream for user $u_{l,k}$ is

(14) $$\gamma_{l,k}^{p}[n]=\frac{|h_{l,k}^{H}[n]w_{p,l,k}[n]{|}^{2}}{\sum_{i=1,i\neq k}^{m_{l}}|h_{l,k}^{H}[n]w_{p,l,i}[n]{|}^{2}+\sigma_{l,k}^{2}}$$

Therefore, the achievable rate of the private stream for user $u_{l,k}$ in time slot n is

(15) $$R_{l,k}^{p}[n]=\log_{2}(1+\gamma_{l,k}^{p}[n]),\forall l,n$$

When $\alpha_{l}[n]=1$, the total communication rate of user $u_{l,k}$ at time slot n is

(16) $$R_{l,k}^{tol}[n]=c_{l,k}[n]+R_{l,k}^{p}[n],\forall ,l,n,k$$

The sum rate of the system over the entire flight period can be expressed as

(17) $$R_{c}^{tol}=\sum_{n=1}^{N}\sum_{l=1}^{L}\sum_{k=1}^{m_{l}}\alpha_{l}[n]R_{l,k}^{tol}[n]$$

### 2.2. Sensing Model

In the UAV-ISAC system considered in this paper, the downlink RSMA waveform simultaneously supports the dual functions of communication and sensing. Specifically, as described above, the transmit signal of the UAV at time slot n can be represented by the vector signal x[n], which is superposed by the cluster common stream and the user-level private streams. This signal carries the users’ communication data while also being used to sense the ground users. Since the sensing capability in the target direction is mainly determined by the transmit array beampattern gain toward that direction, we directly adopt the beampattern gain of the transmit waveform toward the target user $u_{l,k}$, denoted by $\Gamma \left(q_{U}[n],u_{l,k}\right)$, as the sensing-performance metric. A larger beampattern gain implies more sensing energy available in that direction and thus better parameter estimation accuracy. It can be seen that the design of the RSMA beamforming vectors not only determines the multiuser interference management and the communication rate, but also directly affects the spatial energy distribution of the sensing link, which is the key to achieving communication–sensing joint optimization.

Specifically, the transmit beampattern gain from the UAV toward user $u_{l,k}$ can be expressed as

(18) $$\Gamma \left(q_{U}[n],u_{l,k}\right)=E[|a^{H}\left(q_{U}[n],u_{l,k}\right)x[n]{|}^{2}]$$

where $a^{H}\left(q_{U}[n],u_{l,k}\right)$ is the UPA response vector toward user $u_{l,k}$, and x[n] is the transmit signal at time slot n.

Substituting (7) into (18), the beampattern gain in the direction of user $u_{l,k}$ is obtained as

(19) $$\Gamma \left(q_{U}[n],u_{l,k}\right)=|a^{H}\left(q_{U}[n],u_{l,k}\right)w_{c,l}[n]{|}^{2}+\sum_{i=1}^{m_{l}}|a^{H}\left(q_{U}[n],u_{l,k}\right)w_{p,l,k}[n]{|}^{2}$$

For clarity, we denote the first term as the beampattern gain contributed by the common beam, i.e.,

(20) $$\Gamma_{c}\left(q_{U}[n],u_{l,k}\right)=|a^{H}\left(q_{U}[n],u_{l,k}\right)w_{c,l}[n]{|}^{2}$$

The second term is defined as the beampattern gain contributed by the private stream beams

(21) $$\Gamma_{p}\left(q_{U}[n],u_{l,k}\right)=\sum_{i=1}^{m_{l}}|a^{H}\left(q_{U}[n],u_{l,k}\right)w_{p,l,k}[n]{|}^{2}$$

Accordingly, the overall beampattern gain can be written as

(22) $$\Gamma \left(q_{U}[n],u_{l,k}\right)=\Gamma_{c}\left(q_{U}[n],u_{l,k}\right)+\Gamma_{p}\left(q_{U}[n],u_{l,k}\right)$$

To further quantify the contribution of the private streams to sensing, we adopt the composite transmit beampattern gain in the target direction as the sensing-performance metric. Nevertheless, the physical sensing capability of the proposed ISAC system relies on the radar echoes reflected by the ground users, which experience two-way propagation loss. According to the monostatic radar equation, the received echo signal-to-noise ratio is proportional to the transmit beampattern gain and inversely proportional to the fourth power of the target distance. Accordingly, we define the normalized received sensing-gain metric for target user $u_{l,k}\;$ as:

(23) $$\Gamma_{rec}\left(q_{U}[n],u_{l,k}\right)\propto \frac{\Gamma \left(q_{U}[n],u_{l,k}\right)}{d_{l,k}{\left[n\right]}^{4}}$$

In (23), the target radar cross section, carrier wavelength, receive-array gain, matched-filter processing gain, receiver noise power, and system losses are assumed to remain constant during the considered flight period and are therefore absorbed into the sensing threshold. Hence, the adopted metric captures the dominant effects of transmit beamforming and two-way propagation loss on sensing performance. It should be noted that (23) is a simplified sensing-quality metric, while target-dependent RCS variations, clutter, multipath propagation, and detailed receiver processing are beyond the scope of this work.

### 2.3. Optimization Problem

The UAV’s transmit beamforming design, common-rate allocation, trajectory planning, and user scheduling jointly affect the communication and sensing performance of the system. Therefore, over a given flight period, we aim to maximize the average downlink communication rate of the system subject to constraints on the sensing beampattern gain threshold, user rate quality of service (QoS), transmit power, and the UAV mobility dynamics. Consequently, the problem can be formulated as follows:

(24) $$\begin{array}{ll} P0: & \underset{W,C,Q,\alpha }{\max}\frac{1}{N}\sum_{n=1}^{N}\sum_{l=1}^{L}\sum_{k=1}^{m_{l}}\alpha_{l}R_{l,k}^{tol}[n]\\ s.t. & C1:\sum_{l=1}^{L}\alpha_{l}[n]\leq 1,\forall ,n\\ & C2:\alpha_{l}[n]\in \{0,1\},\forall l,n\\ & C3:\frac{\Gamma \left(q_{U}[n],u_{l,k}\right)}{d_{l,k}{\left[n\right]}^{4}}\geq \alpha_{l}[n]\Gamma_{l,k}^{th},\forall l,k,n\\ & C4:c_{l,k}\geq 0,\forall l,k\\ & C5:\sum_{k=1}^{m}c_{l,k}[n]\leq R_{l}^{com}[n],\forall l,n\\ & C6:\sum_{n=1}^{N}\alpha_{l}[n]R_{l,k}^{tol}[n]\geq R_{u}^{th},\forall ,l,k\\ & C7:\sum_{l=1}^{L}\alpha_{l}[n](||w_{c,l}[n]|{|}^{2}+\sum_{k=1}^{m_{l}}||w_{p,l,k}[n]|{|}^{2})\leq P_{\max},\forall n\\ & C8:q_{U}[1]=q_{U}[N]=q_{0}\\ & C9:||q_{U}[n]-q_{U}[n-1]||\leq v_{\max}\delta_{t} \end{array}$$

Here, C1 and C2 ensure that at most one group of users is scheduled for downlink communication and sensing in each time slot, the UAV serves only one user cluster per slot. C3 is the sensing-performance constraint: when cluster l is scheduled at time slot n, the normalized transmit beampattern gain in the directions of all users in that cluster is no less than the prescribed threshold $\Gamma_{l,k}^{th}$, thereby ensuring that the normalized received sensing gain satisfies the prescribed sensing-quality requirement. C4 enforces non-negative common information rate allocation. C5 requires that the sum of the common rates allocated to the users in cluster l does not exceed the decodable common stream rate of that cluster at time slot n, so that all in-cluster users can successfully decode the common stream. C6 is the communication performance constraint: over the entire flight period, the total rate of each user is no less than its minimum rate requirement $R_{u}^{th}$. C7 limits the UAV transmit power in each time slot to not exceed the maximum transmit power $P_{\max}$. C8 requires that the UAV starts and ends at the same location. C9 constrains the UAV displacement between two consecutive time slots.

## 3. Joint Optimization Algorithm

The optimization problem P0 is a mixed-integer non-convex optimization problem in which cluster scheduling, common-rate allocation, common and private beamforming, and UAV trajectory variables are strongly coupled. However, these variables exhibit a natural block structure. When the other variable blocks are fixed, the scheduling subproblem has a linear structure, the rate-allocation and beamforming subproblem can be handled using semidefinite relaxation and successive convex approximation, and the UAV trajectory subproblem can be approximated by a sequence of convex problems. Motivated by this structure, we adopt a block-wise alternating optimization framework and decompose P0 into three subproblems: cluster scheduling, rate allocation and beamforming, and UAV trajectory optimization. The ground-user clustering is performed once before the alternating optimization and is treated as an offline preprocessing step.

### 3.1. Clustering

To efficiently handle the combinatorial complexity inherent in user scheduling for the proposed RSMA system, we employ a two-stage hierarchical optimization strategy. Directly scheduling the K ground users would result in a complex integer programming problem with a search space of $2^{K}$, which is computationally prohibitive. Thus, a static user clustering step is introduced prior to the dynamic scheduling. The rationale for this pre-clustering is two-fold. Firstly, users in close spatial proximity typically possess highly correlated channel characteristics. Grouping them into fixed clusters enables the UAV to transmit common stream beams that effectively mitigate intra-cluster interference and exploit spatial diversity gains. Secondly, pre-clustering reduces the original user-level combinatorial scheduling space to a smaller set of candidate clusters. Although the scheduling subproblem still retains dynamic decision-making, the reduced number of scheduling variables significantly lowers the computational burden of the subsequent alternating optimization procedure.

The proposed procedure follows an agglomerative hierarchical clustering principle, in which spatially adjacent clusters are iteratively merged subject to capacity and distance-related constraints [24]. Based on this principle, we adopt a capacity–diameter-constrained clustering algorithm.

For the capacity limit and whose merged diameter does not exceed a given threshold, the pair that yields the smallest post merge intra-cluster diameter and merge them. If multiple candidate pairs exist, priority is given to the pair with a smaller centroid distance. When there is no feasible cluster pair satisfying the capacity diameter constraints, the iteration terminates and a fixed cluster set ℜ is obtained. Here, the cluster diameter is defined as the maximum Euclidean distance $d_{i,j}$ between any two users’ three dimensional coordinates within the same cluster, which characterizes the spatial spread of the cluster. The detailed procedure is summarized in Algorithm 1.
**Algorithm 1.** Clustering Algorithm**Input:** user coordinates $u_{k};$ capacity limit
m; threshold
$d_{\max}$
**Output:** cluster set.Compute the joint distance $d_{i,j}$ for all user pairs.Initialization: each user forms an individual cluster, i.e., ${ℜ}_{k}=\{k\}$.Iteration: among all cluster pairs that remain feasible after merging, merge the pair with the minimum inter cluster distance, and update the distances between the merged cluster and the other clusters.Terminate when no feasible cluster pair exists.

### 3.2. Scheduling Optimization

Given the beamforming vectors, rate allocation, and UAV trajectory, the subproblem of optimizing the scheduling variables $\alpha_{l}[n]$ can be formulated as

(25) $$\begin{array}{ll} P1: & \underset{\alpha }{\max}\frac{1}{N}\sum_{n=1}^{N}\sum_{l=1}^{L}\sum_{k=1}^{m_{l}}\alpha_{l}[n]R_{l,k}^{tol}[n]\\ s.t. & C1,C2,C3,C6,C7. \end{array}$$

Since the scheduling variable $\alpha_{l}[n]$ is a discrete binary variable, problem P1 is an integer programming problem and cannot be directly solved using convex optimization tools such as CVX. To reduce the computational complexity, we relax the scheduling variables to be continuous by replacing constraint C2 with its interval form

(26) $$\;\tilde{C2}:0\leq \alpha_{l}[n]\leq 1,\forall l,n$$

Then, problem P1 can be written as

(27) $$\begin{array}{ll} P1.1: & \underset{\alpha }{\max}\frac{1}{N}\sum_{n=1}^{N}\sum_{l=1}^{L}\sum_{k=1}^{m_{l}}\alpha_{l}R_{l,k}^{tol}[n]\\ s.t. & C1,\tilde{C2},C3,C6,C7. \end{array}$$

In P1.1, both the objective function and all constraints with respect to $\alpha_{l}[n]$ are linear; hence, P1.1 is a standard linear programming problem and can be solved using tools such as CVX. Let $\alpha_{l}^{*}[n]$ denote the optimal solution of the relaxed problem. For each time slot n, the cluster with the largest $\alpha_{l}^{*}[n]$ is selected as the candidate scheduled cluster. Its scheduling variable is set to one, while the scheduling variables of the remaining clusters are set to zero. This procedure generates a candidate binary scheduling solution, which is then checked against all the original scheduling-related constraints of P1, including C1, C3, C6, and C7. The rounded solution is accepted only if it is feasible and its objective value is no lower than that achieved by the feasible scheduling solution from the previous outer iteration. Otherwise, the previous feasible scheduling solution is retained.

### 3.3. Rate Allocation and Beamforming Optimization

Given the scheduling variables and the UAV trajectory, the subproblem of common-rate allocation and beamforming can be formulated as

(28) $$\begin{array}{ll} P2: & \underset{C,W}{\max}\frac{1}{N}\sum_{n=1}^{N}\sum_{l=1}^{L}\sum_{k=1}^{m_{l}}\alpha_{l}R_{l,k}^{tol}[n]\\ s.t. & C3,C4,C5,C6,C7 \end{array}$$

From (27), it can be observed that the objective function of P2 and constraints C3, C6 and C7 all contain non-convex quadratic terms with respect to the beamforming vectors, and the private rate $R_{l,k}^{p}[n]$ appears in fractional form. Therefore, given the scheduling and trajectory, subproblem P2 is a strongly non-convex joint optimization problem of rate allocation and beamforming. To tackle this, we sequentially apply semidefinite relaxation (SDR) [25], successive convex approximation (SCA) [26], and a maximum-eigenvalue-based penalty strategy [27] to solve P2 and recover near-rank-one beamforming matrices.

We introduce the matrix variables $H_{l,k}[n]=h_{l,k}[n]h_{l,k}^{H}[n]$, $W_{c,l}[n]=w_{c,l}[n]w_{c,l}^{H}[n]$, $W_{p,l,k}[n]=w_{p,l,k}[n]w_{p,l,k}^{H}[n]$ and impose the rank one constraints

(29) $$D2:rank(W_{c,l}[n])=1,rank(W_{p,l,k}[n])=1,\forall l,k,n$$

With these definitions, the quadratic terms of the beamforming vectors can be rewritten as

$$\left||h_{l,k}^{H}[n]w_{c,l}[n]|{|}^{2}=tr(H_{l,k}[n]W_{c,l}[n]\right)\;||h_{l,k}^{H}[n]w_{p,l,k}[n]|{|}^{2}=tr(H_{l,k}[n]W_{p,l,k}[n])$$

Then, the SINR and the achievable rate of the private stream can be rewritten as

(30) $$R_{l,k}^{p}[n]=\log_{2}\frac{\sum_{i=1}^{m_{l}}tr(H_{l,k}[n]W_{p,l,i}[n])+\sigma_{l,k}^{2}}{\sum_{i=1,i\neq k}^{m_{l}}tr(H_{l,k}[n]W_{p,l,i}[n])+\sigma_{l,k}^{2}}$$

Similarly, the beampattern gain term in sensing constraint C3 can be expressed as a linear function of the covariance matrices. Define

(31) $$A_{l,k}[n]=a\left(q_{U}[n],u_{l,k}\right)a^{H}\left(q_{U}[n],u_{l,k}\right)$$

Then, the sensing threshold constraint C3 is equivalently rewritten as

(32) $$C3^{\prime }:\frac{tr(A_{l,k}[n]W_{c,l}[n])+\sum_{i=1}^{m_{l}}tr(A_{l,k}[n]W_{p,l,i}[n])}{d_{l,k}{\left[n\right]}^{4}}\geq \alpha_{l}[n]\Gamma_{l,k}^{th}$$

Expanding the private rate term in C6 into its explicit fractional form, C6 can be rewritten as

(33) $$C6^{\prime }:\sum_{n=1}^{N}\alpha_{l}[n]\left(c_{l,k}[n]+\log_{2}\frac{\sum_{i=1}^{m_{l}}tr(H_{l,k}[n]W_{p,l,i}[n])+\sigma_{l,k}^{2}}{\sum_{i=1,i\neq k}^{m_{l}}tr(H_{l,k}[n]W_{p,l,i}[n])+\sigma_{l,k}^{2}}\right)\geq R_{u}^{th},\forall ,l,k$$

Meanwhile, the power constraint C7 is rewritten as

(34) $$C7^{\prime }:\sum_{l=1}^{L}\alpha_{l}[n](tr(W_{c,l}[n])+\sum_{k=1}^{m_{l}}tr(W_{p,l,k}[n]))\leq P_{\max},\forall n$$

We further impose positive semidefinite constraints on the matrix variables

(35) $$D1:W_{c,l}[n]\geq 0,W_{p,l,k}[n]\geq 0,\forall ,l,k,n.$$

Based on the above variable transformations, problem P2 can be equivalently converted into the following matrix form problem P2.1

(36) $$\begin{array}{ll} P2.1: & \underset{C,W}{\max}\frac{1}{N}\sum_{n=1}^{N}\sum_{l=1}^{L}\sum_{k=1}^{m_{l}}\alpha_{l}[n](c_{l,k}[n]+R_{l,k}^{p}[n])\\ s.t. & C3^{\prime },C4,C5,C6^{\prime },C7^{\prime },D1,D2, \end{array}$$

It can be seen that although SDR linearizes the quadratic terms, $R_{l,k}^{p}[n]$ is still in a difference in logarithms form, and the rank constraint D2 remains. Hence, P2.1 is still a non-convex semidefinite programming problem.

To further handle the non-convexity of the private rate term, we introduce the auxiliary variables $\phi_{l,k}[n]=\sum_{i=1}^{m_{l}}tr(H_{l,k}[n]W_{p,l,i}[n])+\sigma_{l,k}^{2}$, $\varphi_{l,k}[n]=\sum_{i=1,i\neq k}^{m_{l}}tr(H_{l,k}[n]W_{p,l,i}[n])+\sigma_{l,k}^{2}$.

Then we have

(37) $$R_{l,k}^{p}[n]=\log_{2}\phi_{l,k}[n]-\log_{2}\varphi_{l,k}[n]$$

At the jth iteration, we perform the first order Taylor expansion of the concave function $\log_{2}\varphi_{l,k}[n]$ at the previous point $\varphi_{l,k}^{\left(j\right)}[n]$, yielding

(38) $$\log_{2}\varphi_{l,k}[n]\leq \log_{2}\varphi_{l,k}^{\left(j\right)}[n]+\frac{1}{\ln2}\frac{\varphi_{l,k}[n]-\varphi_{l,k}^{\left(j\right)}[n]}{\varphi_{l,k}^{\left(j\right)}[n]}$$

Accordingly, a lower bound of $R_{l,k}^{p}[n]$ is obtained as

(39) $${\hat{R}}_{l,k}^{p}[n]=\log_{2}\phi_{l,k}[n]-\left[\log_{2}\varphi_{l,k}^{\left(j\right)}[n]+\frac{1}{\ln2}\frac{\varphi_{l,k}[n]-\varphi_{l,k}^{\left(j\right)}[n]}{\varphi_{l,k}^{\left(j\right)}[n]}\right]$$

Since $\log_{2}\phi_{l,k}[n]$ is concave and the linear term is also concave, ${\hat{R}}_{l,k}^{p}[n]$ is a concave lower bound, which guarantees that the resulting objective is concave. We replace the true private rate by this lower bound and introduce an auxiliary variable $\gamma_{l,k}[n]$ for the QoS constraint C6’. Constraint E1 ensures that $\gamma_{l,k}[n]$ does not exceed the current iteration lower bound ${\hat{R}}_{l,k}^{p}[n]$, and thus E2 guarantees that substituting the true rate in E2 will not violate the QoS requirement:

(40) $$E1:\;\gamma_{l,k}[n]\leq {\hat{R}}_{l,k}^{p}[n],\;\forall l,k,n$$

(41) $$E2:\;\sum_{n=1}^{N}\alpha_{l}[n](c_{l,k}[n]+\gamma_{l,k}[n])\geq R_{l,k}^{th},\forall l,k.$$

Accordingly, the convex approximation subproblem to be solved at the j th iteration can be constructed as

(42) $$\begin{array}{ll} P2.2: & \underset{C,W,\gamma }{\max}\frac{1}{N}\sum_{n=1}^{N}\sum_{l=1}^{L}\sum_{k=1}^{m_{l}}\alpha_{l}[n](c_{l,k}[n]+\gamma_{l,k}[n])\\ s.t. & C3^{\prime },C4,C5,C7^{\prime },D1,E1,E2. \end{array}$$

If we temporarily ignore the rank constraint D2, problem P2.2 reduces to a standard semidefinite program and can be efficiently solved using convex optimization tools such as CVX, thereby yielding the beamforming and rate updates at the current iteration point.

However, the SDR does not enforce $W_{c,l}[n]$ and $W_{p,l,k}[n]$ to be rank one, and the direct solution of P2.2 typically results in high-rank matrices. To gradually recover a near rank one structure from the relaxed solution, we further adopt a penalty function strategy based on the maximum eigenvalue to handle the rank constraint D2.

Let $\lambda_{c,l}^{\left(j\right)}[n]$ and $u_{c,l}^{\left(j\right)}[n]$ denote the maximum eigenvalue of $W_{c,l}[n]$ and its corresponding unit norm eigenvector at the jth iteration, respectively. Similarly, let $\lambda_{p,l,k}^{\left(j\right)}[n]$ and $u_{p,l,k}^{\left(j\right)}[n]$ denote the maximum eigenvalue of $W_{p,l,k}[n]$ and its corresponding eigenvector. Then,

(43) $$\begin{array}{l} \lambda_{c,l}^{\left(j\right)}[n]=(u_{c,l}^{\left(j\right)}{\left[n\right]}^{H}W_{c,l}[n]u_{c,l}^{\left(j\right)}[n])\\ \lambda_{p,l,k}^{\left(j\right)}[n]=(u_{p,l,k}^{\left(j\right)}{\left[n\right]}^{H}W_{p,l,k}[n]u_{p,l,k}^{\left(j\right)}[n]) \end{array}$$

Intuitively, when $W_{c,l}[n]$ is rank one, its trace equals its maximum eigenvalue. Similarly, the rank one property of $W_{p,l,k}[n]$ can be approximately characterized by $tr(W_{p,l,k}[n])-\lambda_{p,l,k}^{\left(j\right)}[n]\approx 0$.

Based on this, we introduce a penalty factor $\rho_{1}>0$ and penalize the above relaxation gaps in the objective function, which leads to the penalized optimization problem

(44) $$\begin{array}{l} P2.3:\underset{C,W,\gamma }{\max}\frac{1}{N}\sum_{n=1}^{N}\sum_{l=1}^{L}\sum_{k=1}^{m_{l}}\alpha_{l}[n](c_{l,k}[n]+\gamma_{l,k}[n])\\ -\rho_{1}\sum_{n,l}\left(tr(W_{c,l}[n])-{\left(u_{c,l}^{\left(j\right)}[n]\right)}^{H}W_{c,l}[n]u_{c,l}^{\left(j\right)}[n]\right)\\ -\rho_{1}\sum_{n,l,k}\left(tr(W_{p,l,k}[n])-{\left(u_{p,l,k}^{\left(j\right)}[n]\right)}^{H}W_{p,l,k}[n]u_{p,l,k}^{\left(j\right)}[n]\right)\\ s.t.\;C3^{\prime },C4,C5,C7^{\prime },D1,E1,E2. \end{array}$$

It is worth noting that, given the eigenvectors $u_{c,l}^{\left(j\right)}[n]$ and $u_{p,l,k}^{\left(j\right)}[n]$ from the previous iteration, P2.3 remains a convex semidefinite programming problem with respect to $W_{c,l}[n]$, $W_{p,l,k}[n]$, C, and γ. Therefore, it can still be solved using CVX. The detailed procedure is summarized in Algorithm 2.
**Algorithm 2.** SDR-Based Common-Rate Allocation and Beamforming Optimization Algorithm**Input:** $\Gamma_{l,k}^{th}$, $R_{u}^{th}$, $P_{\max}$, Q, α, $c_{l,k}^{\left(0\right)}[n]$, $W_{c,l}{\left[n\right]}^{\left(0\right)}$, $W_{p,l,k}{\left[n\right]}^{\left(0\right)}$, $\rho_{1}$, τ, $J_{\max}$**Initialization:** Set j=0. Obtain $u_{c,l}{\left[n\right]}^{\left(0\right)}$ and $u_{p,l,k}{\left[n\right]}^{\left(0\right)}$ as the principal eigenvectors of $W_{c,l}{\left[n\right]}^{\left(0\right)}$ and $W_{p,l,k}{\left[n\right]}^{\left(0\right)}$ respectively. Compute and record the initial objective value $opt^{\left(0\right)}$.
**Repeat**      1.$\mathrm{With}\;u_{c,l}{\left[n\right]}^{\left(j\right)}$ and $u_{p,l,k}{\left[n\right]}^{\left(j\right)}$ fixed, solve problem P2.3 using CVX to obtain $W_{c,l}{\left[n\right]}^{\left(j+1\right)}$, $W_{p,l,k}{\left[n\right]}^{\left(j+1\right)}$, and the corresponding rate variables.      2.$\mathrm{Perform}\;\mathrm{eigen}\;\mathrm{decomposition}\;\mathrm{of}\;W_{c,l}{\left[n\right]}^{\left(j+1\right)}$ and $W_{p,l,k}{\left[n\right]}^{\left(j+1\right)}$, take the eigenvectors corresponding to the maximum eigenvalues, and update $u_{c,l}{\left[n\right]}^{\left(j+1\right)}$ and $u_{p,l,k}{\left[n\right]}^{\left(j+1\right)}$.      3.$\mathrm{Compute}\;\mathrm{the}\;\mathrm{new}\;\mathrm{objective}\;\mathrm{value}\;opt^{\left(j+1\right)}$ and set j=j+1. If $|opt^{\left(j+1\right)}-opt^{\left(j\right)}|\leq \tau$ or $j\geq J_{\max}$, terminate the iteration.**Output:** the converged $W_{c,l}^{*}[n]$, $W_{p,l,k}^{*}[n]$, and the corresponding rate variables.

### 3.4. UAV Trajectory Optimization

Given the cluster scheduling, common-rate allocation, and beamforming vectors, the UAV trajectory optimization subproblem can be formulated as

(45) $$\begin{array}{l} P3:\underset{Q}{\max}\frac{1}{N}\sum_{n=1}^{N}\sum_{l=1}^{L}\sum_{k=1}^{m_{l}}\alpha_{l}R_{l,k}^{tol}[n]\\ s.t.\;C3,C6,C8,C9 \end{array}$$

To simplify the subsequent derivations, we define the squared distance between the UAV and user k in cluster l at time slot n as $z_{l,k}[n]=d_{l,k}^{2}[n]$.

Under the adopted free space path loss model, the large scale channel gain is proportional to $1/d_{l,k}^{2}[n]$. Meanwhile, the array directional gain $a\left(•\right)$ also varies with the UAV trajectory. Therefore, both the communication rate and the sensing gain are nonlinearly coupled with $q_{U}[n]$ in a fractional form, which makes the problem difficult to solve directly. To address this, we employ the successive convex approximation method.

At the r th iteration, given the trajectory $q_{U}^{\left(r-1\right)}[n]$ from the previous iteration, we linearize the array directional gain and treat the direction dependent term as a constant [22]. Specifically, we define

(46) $$a_{l,k}^{\left(r-1\right)}[n]≜a\left(q_{U}^{\left(r-1\right)}[n],u_{l,k}\right)$$

Then, in the current iteration we have the approximation

(47) $$\underset{C_{A,l,k}^{\left(r-1\right)}[n]}{\underset{︸}{\Gamma \left(q_{U}[n],u_{l,k}\right)\approx |a^{\left(r-1\right)H}[n]w_{c,l}[n]{|}^{2}+\sum_{i=1}^{m}\left|a^{\left(r-1\right)H}[n]w_{p,l,i}[n\right]{|}^{2}}}$$

Here, $C_{A,l,k}^{\left(r-1\right)}[n]$ depends only on the previous trajectory and the beamforming vectors, and can thus be regarded as a constant. Accordingly, the sensing threshold constraint C3 in the r th iteration can be equivalently rewritten as an upper bound with respect to the squared distance. Since the scheduling variables are fixed and binary in the trajectory optimization subproblem, the sensing constraint only needs to be enforced for the scheduled cluster–time-slot pairs.

(48) $$CS1:\;d_{l,k}{\left[n\right]}^{2}\leq \sqrt{\frac{C_{A,l,k}^{\left(r-1\right)}[n]}{\Gamma_{l,k}^{th}}},\forall l,k,n\;with\;\alpha_{l}[n]=1;$$

This is a convex quadratic constraint with respect to $d_{l,k}^{2}[n]$.

Moreover, from (15) the private stream rate can be obtained, where both the numerator and denominator of $\gamma_{l,1}^{p}[n]$ vary with the path loss. As a result, the private rate can finally be expressed as a function of the squared distance $z_{l,k}[n]=d_{l,k}^{2}[n]$. Using the SDR-based matrix notation, it can be written as

(49) $$R_{l,k}^{p}[n]=f_{l,k}\left(z_{l,k}[n]\right)=\log_{2}(1+\frac{A_{l,k}^{\left(r-1\right)}[n]}{B_{l,k}^{\left(r-1\right)}[n]+d_{l,k}^{2}[n]\sigma_{l,k}^{2}})$$

Here, $A_{l,k}^{\left(r-1\right)}[n]$ and $B_{l,k}^{\left(r-1\right)}[n]$ are computed from the current iteration trajectory $q_{U}^{\left(r-1\right)}[n]$ and the given beams $w_{c,l}[n]$ and $w_{p,l,k}[n]$, and are constants within this trajectory optimization step. It is easy to verify that $f_{l,k}\left(z\right)$ is a convex function for z>0.

To obtain a convex approximation for the QoS constraint, we construct a global underestimator (affine lower bound) of this convex function at the current point. At the r th SCA iteration, we perform the first order Taylor expansion of $f_{l,k}\left(z\right)$ at the previous squared distance point $z_{l,k}^{\left(r-1\right)}={\left(d_{l,k}^{\left(r-1\right)}[n]\right)}^{2}$, yielding

(50) $${\hat{R}}_{l,k}^{p}[n]=f_{l,k}\left(z_{l,k}^{\left(r-1\right)}[n]\right)+f_{l,k}^{\prime }\left(z_{l,k}^{\left(r-1\right)}[n]\right)\left(z_{l,k}[n]-z_{l,k}^{\left(r-1\right)}[n]\right)\leq R_{l,k}^{p}[n]$$

Therefore, ${\hat{R}}_{l,k}^{p}[n]$ is an affine lower bound of $R_{l,k}^{p}[n]$. Replacing the true private rate with this lower bound leads to a convex approximation of the QoS constraint C6:

(51) $$CS2:\;\sum_{n=1}^{N}\alpha_{l}[n]\left(c_{l,k}[n]+{\hat{R}}_{l,k}^{p}[n]\right)\geq R_{u}^{th},\forall l,k$$

Meanwhile, the same lower bound ${\hat{R}}_{l,k}^{p}[n]$ is also used in the objective function to approximate the true rate, ensuring that the objective value is non-decreasing during the iterative procedure.

In summary, at the r th SCA iteration, the trajectory optimization problem can be approximated as

(52) $$\begin{array}{ll} P3.2: & \underset{Q,z_{l,k}[n]}{\max}\frac{1}{N}\sum_{n=1}^{N}\sum_{l=1}^{L}\sum_{k=1}^{m}\alpha_{l}[n]\left(c_{l,k}[n]+{\hat{R}}_{l,k}^{p}[n]\right)\\ s.t. & CS1,CS2,C8,C9\\ & CS3:z_{l,k}[n]\geq d_{l,k}^{2}[n],\forall ,l,n,k \end{array}$$

Here, ${\hat{R}}_{l,k}^{p}[n]$ is an affine approximation function of $z_{l,k}[n]$, with coefficients computed based on the trajectory from the previous iteration $q_{U}^{\left(r-1\right)}$. For CS3, since $R_{l,k}^{p}[n]$ is monotonically decreasing with $z_{l,k}[n]$, P3.2 becomes a standard convex optimization problem and can be efficiently solved using tools such as CVX. The detailed procedure is summarized in Algorithm 3.
**Algorithm 3.** SCA UAV Trajectory Optimization Algorithm**Input:** $\Gamma_{l,k}^{th}$, $R_{u}^{th}$, $P_{\max}$, α, C, W, $q_{U}^{\left(0\right)}$, $R_{\max}$, ε.
**Initialization:** Set the iteration index r=0. Using $q_{U}^{\left(0\right)}$, compute the constant terms $C_{A,l,k}^{\left(0\right)}[n]$, $A_{l,k}^{\left(0\right)}[n]$, and $B_{l,k}^{\left(0\right)}[n]$, and then obtain the initial objective value $opt^{\left(0\right)}$.
**Repeat:**      1.Based on the current trajectory $q_{U}^{\left(r\right)}$, compute the path loss and directional gain, up date the constant terms $C_{A,l,k}^{\left(r\right)}[n]$, $A_{l,k}^{\left(r\right)}[n]$, and $B_{l,k}^{\left(r\right)}[n]$, and construct the affine lower bound ${\hat{R}}_{l,k}^{p}[n]$ at $z_{l,k}^{\left(r\right)}={\left(d_{l,k}^{\left(r\right)}[n]\right)}^{2}$.      2.Solve the convex problem P3.2 in CVX to obtain the new trajectory and variables $z_{l,k}^{\left(r+1\right)}[n]$, and compute the corresponding objective value $opt^{\left(r+1\right)}$.      3.If ${∥q_{U}^{\left(r+1\right)}-q_{U}^{\left(r\right)}∥}_{F}\leq \varepsilon$ or $r\geq R_{\max}$, terminate; set r=r+1 and go back to Step 1.**Output:** the converged UAV trajectory $Q^{*}$.

### 3.5. BCD Iterative Optimization Algorithm

In summary, the ground users are first clustered in an offline preprocessing stage, after which the original problem is decomposed into three variable blocks: cluster scheduling, common-rate allocation and beamforming, and UAV trajectory optimization. These variable blocks are sequentially updated within the outer alternating optimization procedure to obtain a converged feasible solution. The detailed procedure is summarized in Algorithm 4.
**Algorithm 4.** BCD Joint Optimization Algorithm**Input:** initial scheduling $A^{\left(0\right)}$, initial common rate allocation $C^{\left(0\right)}$, initial beamforming $W^{\left(0\right)}$, initial UAV trajectory, stopping threshold *ε*, and maximum number of iterations $T_{\max}$
According to the ground user location information, perform user clustering and obtain the fixed cluster set ℜ.Initialize the global iteration index t=0 and compute the initial objective value $opt^{\left(0\right)}$.Repeat:Given $C^{\left(t\right)}$, $W^{\left(t\right)}$, and $Q^{\left(t\right)}$, solve P1 to obtain $A^{\left(t+1\right)}$.Given $A^{\left(t+1\right)}$ and $Q^{\left(t\right)}$, invoke Algorithm 2 to obtain $C^{\left(t+1\right)}$ and $W^{\left(t+1\right)}$.Given $A^{\left(t+1\right)}$, $C^{\left(t+1\right)}$, and $W^{\left(t+1\right)}$, invoke Algorithm 3 to obtain $Q^{\left(t+1\right)}$.Compute the updated system objective value $opt^{\left(t+1\right)}$ based on $A^{\left(t+1\right)}$, $C^{\left(t+1\right)}$, $W^{\left(t+1\right)}$, and $Q^{\left(t+1\right)}$.If $∥opt^{\left(t+1\right)}-opt^{\left(t\right)}∥\leq \varepsilon_{in}$ or $t+1\geq T_{\max}$, stop; otherwise set t=t+1 and continue.**Output:** $A^{*}$, $C^{*}$, $W^{*}$ and $Q^{*}$.

#### 3.5.1. Convergence Analysis

Let $\left(\alpha^{\left(j\right)},C^{\left(j\right)},W^{\left(j\right)},Q^{\left(j\right)}\right)$ denote the scheduling variables, common-rate allocation, beamforming coefficients, and UAV trajectory obtained after the jth alternating iteration of the proposed algorithm, and let the objective function of problem P0 be denoted by f(α,C,W,Q). Since the cluster set R remains unchanged throughout the entire optimization process, Algorithm 4 updates only three groups of variables sequentially in each alternating iteration. The impact of each update step on the objective function is analyzed as follows.

Given $C^{\left(j\right)}$, $W^{\left(j\right)}$ and $Q^{\left(j\right)}$, we optimally solve the scheduling subproblem P1 to obtain $\alpha^{\left(j+1\right)}$. This update maximizes the original objective with the other variables fixed; hence,

(53) $$f(\alpha^{\left(j+1\right)},C^{\left(j\right)},W^{\left(j\right)},Q^{\left(j\right)})\geq f^{\left(j\right)}$$

Given $\alpha^{\left(j+1\right)}$ and $Q^{\left(j\right)}$, the beamforming and rate subproblem P2 is solved via SCA and SDR. In each SCA iteration, an intermediate problem is constructed using a tight lower bound of the rate, so that the obtained optimal value is non-decreasing. Consequently, after obtaining $C^{\left(j+1\right)}$ and $W^{\left(j+1\right)}$, we have

(54) $$f(\alpha^{\left(j+1\right)},C^{\left(j+1\right)},W^{\left(j+1\right)},Q^{\left(j\right)})\geq f(\alpha^{\left(j+1\right)},C^{\left(j\right)},W^{\left(j\right)},Q^{\left(j\right)})$$

Given $\alpha^{\left(j+1\right)}$, $C^{\left(j+1\right)}$, and $W^{\left(j+1\right)}$, the trajectory subproblem P3 is solved by SCA. Similarly, a rate lower bound is used to build a convex approximation, ensuring that the objective value does not decrease in each iteration. After obtaining $Q^{\left(j+1\right)}$, it holds that

(55) $$f(\alpha^{\left(j+1\right)},C^{\left(j+1\right)},W^{\left(j+1\right)},Q^{\left(j+1\right)})\geq f(\alpha^{\left(j+1\right)},C^{\left(j+1\right)},W^{\left(j+1\right)},Q^{\left(j\right)})$$

Combining the three block-update steps, the rounded scheduling solution is accepted only when it is feasible and does not decrease the objective value; otherwise, the previous feasible scheduling solution is retained. Therefore, the objective-value sequence generated in the outer iterations is monotonically non-decreasing. Since the transmit power is bounded and the achievable communication rate is finite, the objective-value sequence is upper bounded and thus converges to a finite value. However, due to the binary scheduling variables and the non-convex coupling among the continuous variables, the proposed algorithm does not guarantee convergence to a globally optimal solution. The obtained solution should therefore be regarded as an initialization-dependent converged feasible solution.

#### 3.5.2. Complexity Analysis

The overall computational complexity of the proposed alternating optimization algorithm is primarily governed by three subproblems within each outer iteration. Let $I_{0}$ denote the number of outer BCD iterations.

First, the scheduling subproblem P1 is a standard linear programming problem, whose complexity is approximately $O({\left(NL\right)}^{3})$.

Second, the beamforming and rate allocation subproblem P2 is solved via SDR. In each inner SCA iteration, solving the convex SDP for the NL time-slot-cluster pairs yields a complexity of $O(NLM^{3.5})$. Let $I_{1}$ denote the average number of inner SCA iterations required for P2, resulting in a total complexity of $O(I_{1}NLM^{3.5})$.

Third, the trajectory optimization subproblem P3 is addressed by SCA, where each inner iteration involves solving a convex SOCP. Let $I_{2}$ denote the average number of inner SCA iterations for P3. The corresponding complexity is therefore $O(I_{2}{\left(NLm\right)}^{3})$.

Consequently, the overall worst-case computational complexity of the proposed joint optimization algorithm can be expressed as: $O\{I_{0}[({\left(NL\right)}^{3})+(I_{1}NLM^{3.5})+(I_{2}{\left(NLm\right)}^{3})]\}$.

## 4. Numerical Results

This paper uses MATLAB (v2024b) to simulate the proposed joint optimization algorithm. We consider a rectangular area of 800 m×800 m, where the system contains 16 ground users whose horizontal locations are independently and uniformly distributed over the region. The UAV flies at a fixed altitude of H=100 m. The UAV flight duration is T=50 s, which is divided into N=100 equal time slots with length $\delta_{t}=0.5\;s$. The starting and ending positions of the UAV are the same. The sensing threshold is expressed in decibels relative to the reference sensing-gain level $\Gamma_{ref}$. In the simulations, $\Gamma_{ref}=5\times 10^{-10}$. Therefore, 0 dB corresponds to $\Gamma^{th}=\Gamma_{ref}$, rather than zero sensing gain. Unless otherwise specified, the simulation parameters are listed in Table 2.

To evaluate the performance of the proposed algorithm, we compare it with four benchmark schemes:

1. Fix Q-RSMA: The UAV trajectory is not optimized; only user scheduling, the UAV transmit beamforming vectors, and the common-rate allocation are optimized.
2. Beam-NOMA: With the same trajectory and scheduling strategy as in this paper, the transmit signal is optimized using NOMA.
3. Beam-SDMA: With the same trajectory and scheduling strategy as in this paper, the transmit signal is optimized using SDMA.
4. Algorithm in [23]: A time division structure is adopted, and scheduling, power, and trajectory are alternately optimized; however, sensing and communication are performed in separate time slots, and the common and private beamforming is not optimized.

**Figure 2:**
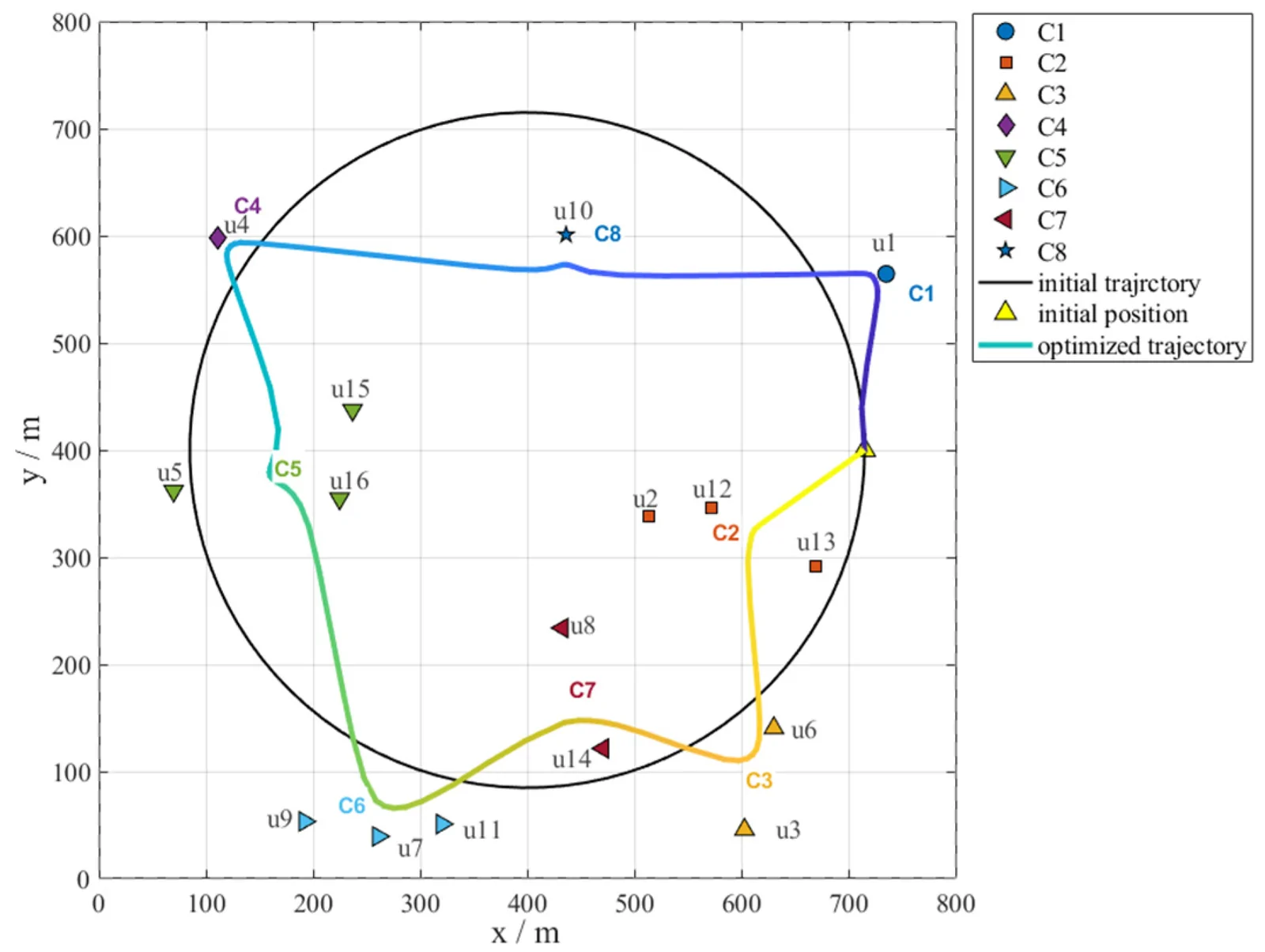
UAV trajectory optimization.

Figure 2 compares the UAV trajectories before and after optimization. It can be observed that, compared with the initial circular trajectory, the optimized trajectory actively steers toward several user clusters during the flight. Under the constraints that the starting and ending points coincide and that the speed constraints are satisfied, the UAV moves closer to the cell edge users and the regions with poorer channel conditions, thereby improving both the system communication rate and sensing performance.

**Figure 3:**
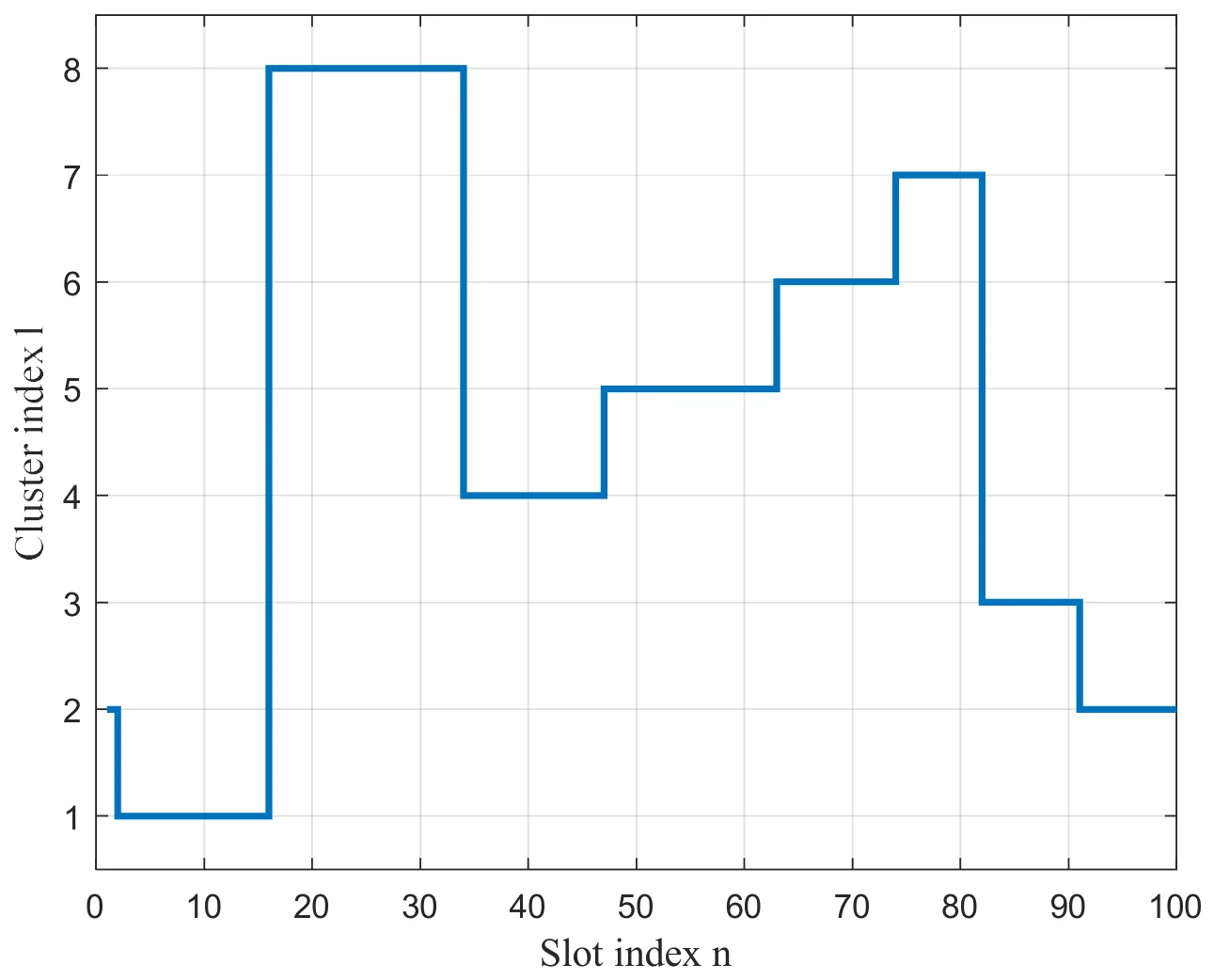
Cluster scheduling results.

Figure 3 shows the cluster scheduling results over all time slots. It can be seen that only one cluster is scheduled for downlink transmission in each time slot. As the UAV moves along its trajectory and approaches different clusters, the scheduled cluster index changes in a stepwise manner: the UAV serves the same cluster for a consecutive set of time slots and then switches to the next cluster. When the UAV is close to a given cluster, it concentrates data transmission to the users in that cluster to better exploit favorable channel conditions and beampattern gains. Meanwhile, by switching clusters at appropriate times, the rate and sensing constraints of users in all clusters are guaranteed.

**Figure 4:**
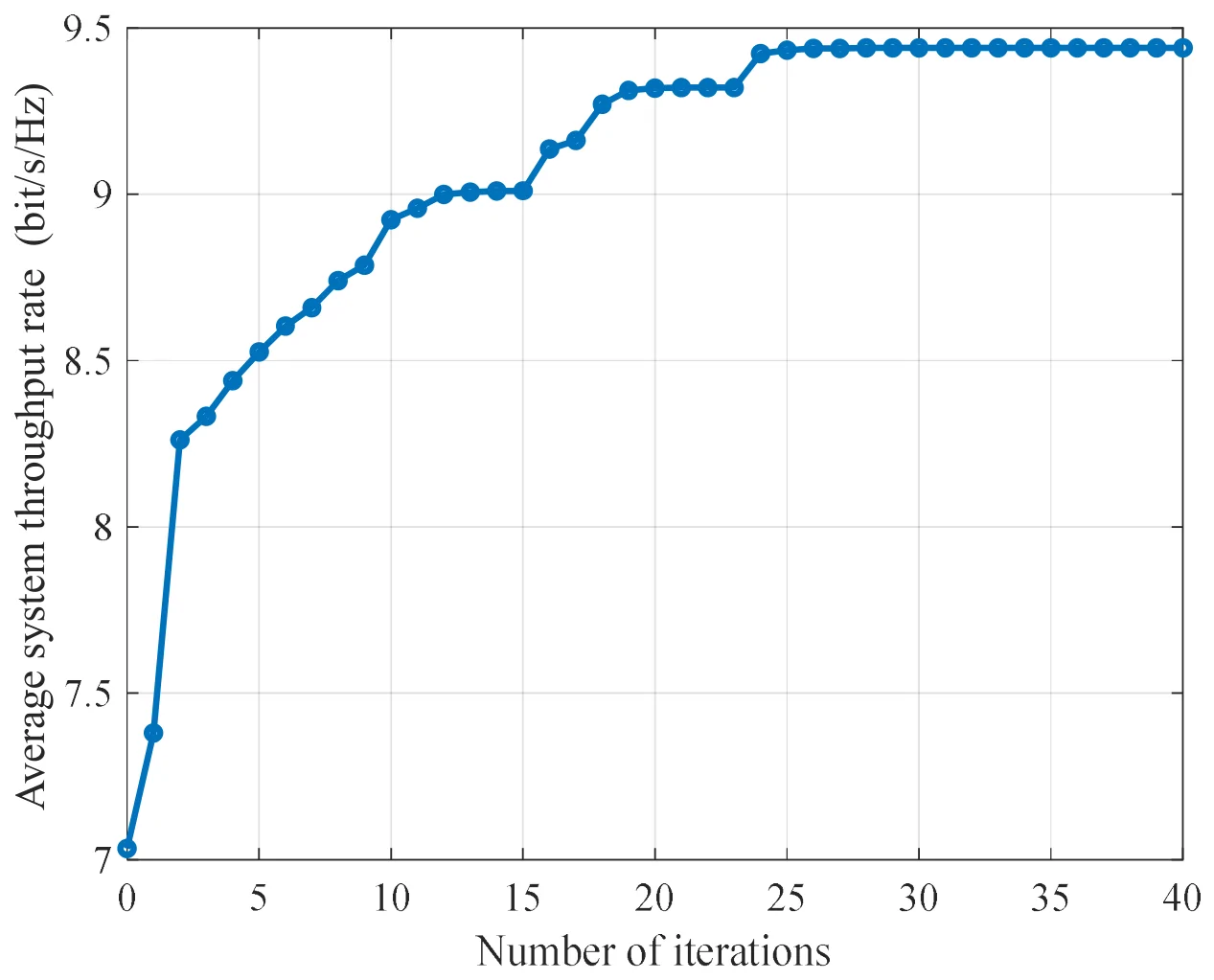
Convergence of the joint optimization algorithm.

Figure 4 illustrates the convergence behavior of the proposed alternating optimization algorithm. The objective value increases rapidly during the first few outer iterations and then improves gradually with smaller increments, becoming stable after approximately 25–30 iterations. In each outer iteration, the rounded scheduling solution is accepted only when it is feasible and does not decrease the objective value; otherwise, the previous feasible scheduling solution is retained. The rate-allocation and beamforming variables and the UAV trajectory are subsequently updated through their corresponding convex surrogate problems. Therefore, the numerical trend in Figure 4 is consistent with the bounded non-decreasing objective-value sequence discussed in Section 3.5.1.

**Figure 5:**
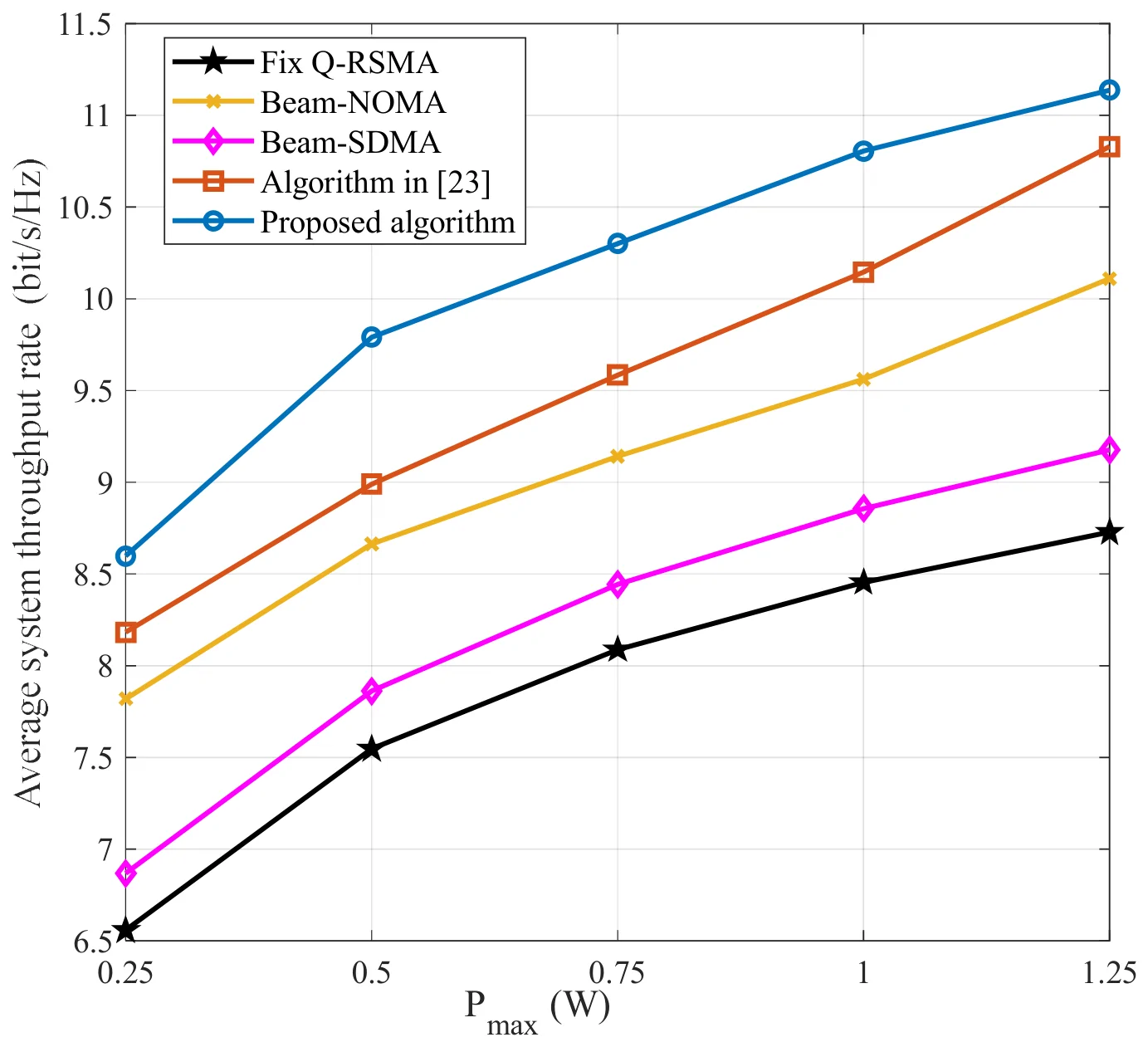
Comparison of different algorithms [23].

Figure 5 presents the comparison results of different algorithms in terms of the average system rate versus the maximum transmit power. It can be observed that, as the maximum transmit power increases, the achievable rates of all algorithms grow monotonically. However, over the entire power range, the proposed algorithm consistently outperforms the other schemes by a clear margin, whereas Fix Q-RSMA always yields the lowest rate. Among the non-RSMA baselines, NOMA is only slightly better than SDMA. Since the algorithm in [23] jointly optimizes the transmit power and the UAV trajectory, it performs better than the other baselines; nevertheless, constrained by the time division operation between sensing and communication and the lack of joint design of common and private beams, it still remains significantly inferior to the proposed joint optimization scheme. The fundamental reason for the above performance differences is that RSMA can handle multiuser interference more effectively by decomposing messages into a common stream and private streams. Moreover, the proposed algorithm further coordinates the UAV position, beam pointing, and power allocation across both spatial and temporal domains, thereby aligning the service direction with the users’ geometric distribution. This not only fully exploits the spatial degrees of freedom provided by the antenna array, but also leverages the trajectory flexibility to enhance link quality. In contrast, schemes that optimize only power or design beams under a fixed trajectory cannot simultaneously balance interference suppression and multiuser multiplexing. Therefore, the proposed algorithm achieves the best performance in jointly exploiting spatial and power resources and improving the system throughput.

**Figure 6:**
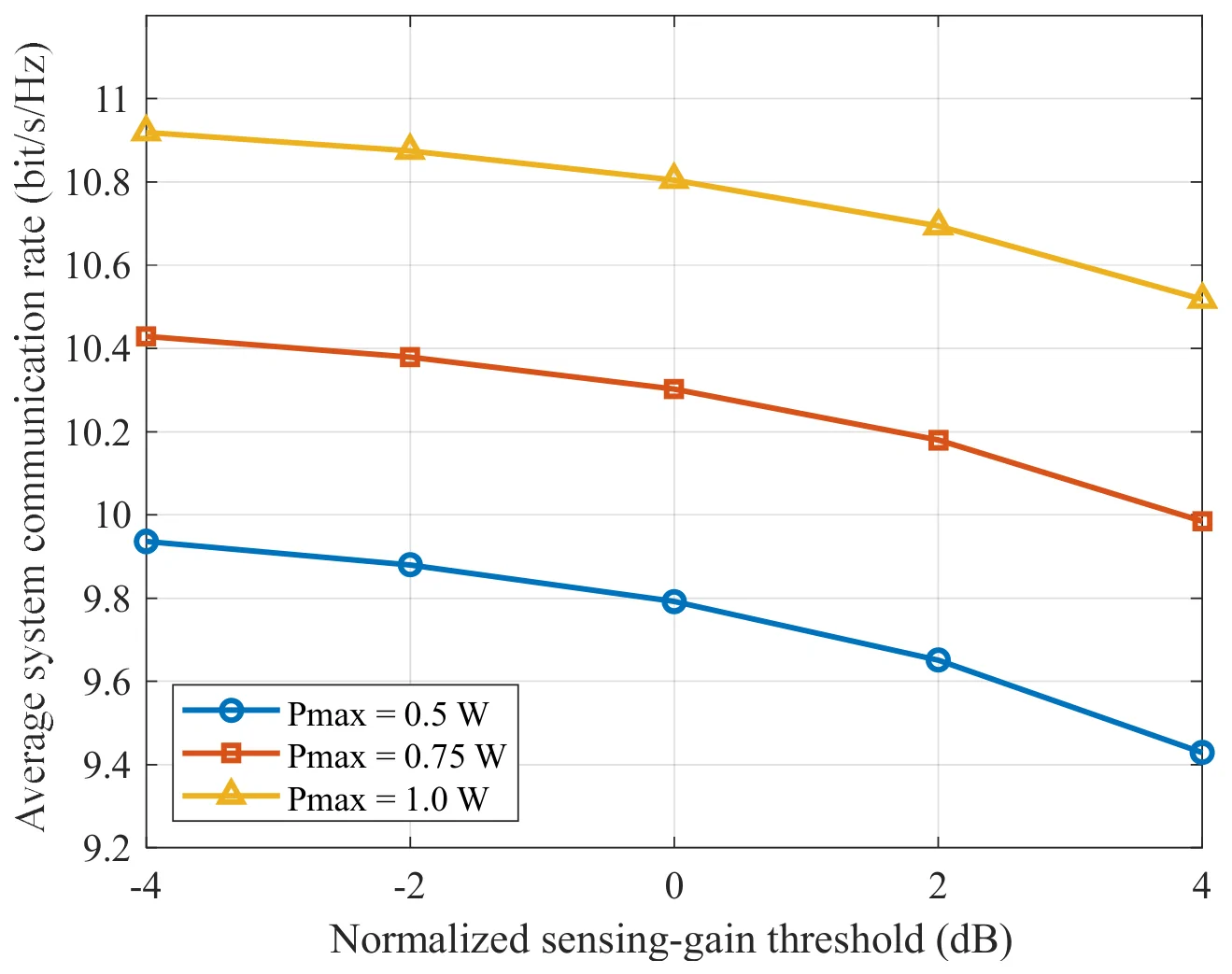
Average system communication rate versus the normalized sensing-gain threshold.

Figure 6 shows the average system communication rate versus the normalized sensing-gain threshold under different maximum transmit-power budgets. The average communication rate decreases as the sensing-gain threshold increases. This is because a more stringent sensing requirement restricts the feasible beamforming and UAV trajectory design space and allocates more transmission resources to sensing, thereby reducing the resources available for communication. Moreover, a larger maximum transmit power consistently improves the communication performance and alleviates the rate degradation caused by the increased sensing requirement. These results clearly demonstrate the communication–sensing tradeoff in the proposed RSMA-enabled UAV-ISAC system.

**Figure 7:**
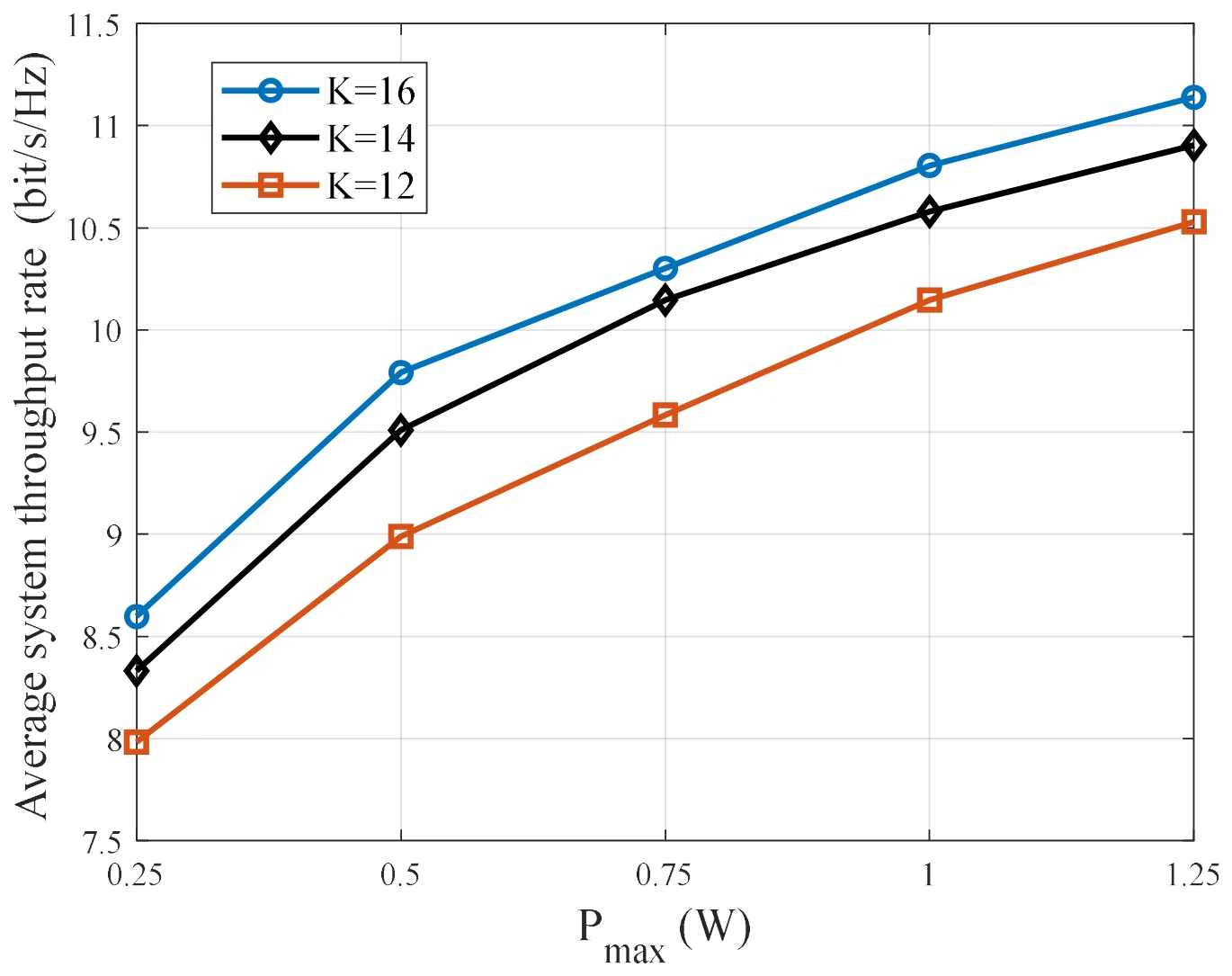
Average system communication rate versus the number of users.

Figure 7 shows the curves of the average system communication rate versus the transmit power for different numbers of users. It can be observed that, as the transmit power increases, all curves increase monotonically, since higher power yields a larger effective signal to interference-plus-noise ratio, thereby improving the achievable rates of both the common stream and the private streams. As the number of users increases, the curves shift upward overall, indicating that under the proposed cluster scheduling and RSMA beamforming joint design, the system can exploit the multiuser diversity and spatial reuse gains brought by more users, and thus maintains a relatively high sum rate even when the user scale expands.

**Figure 8:**
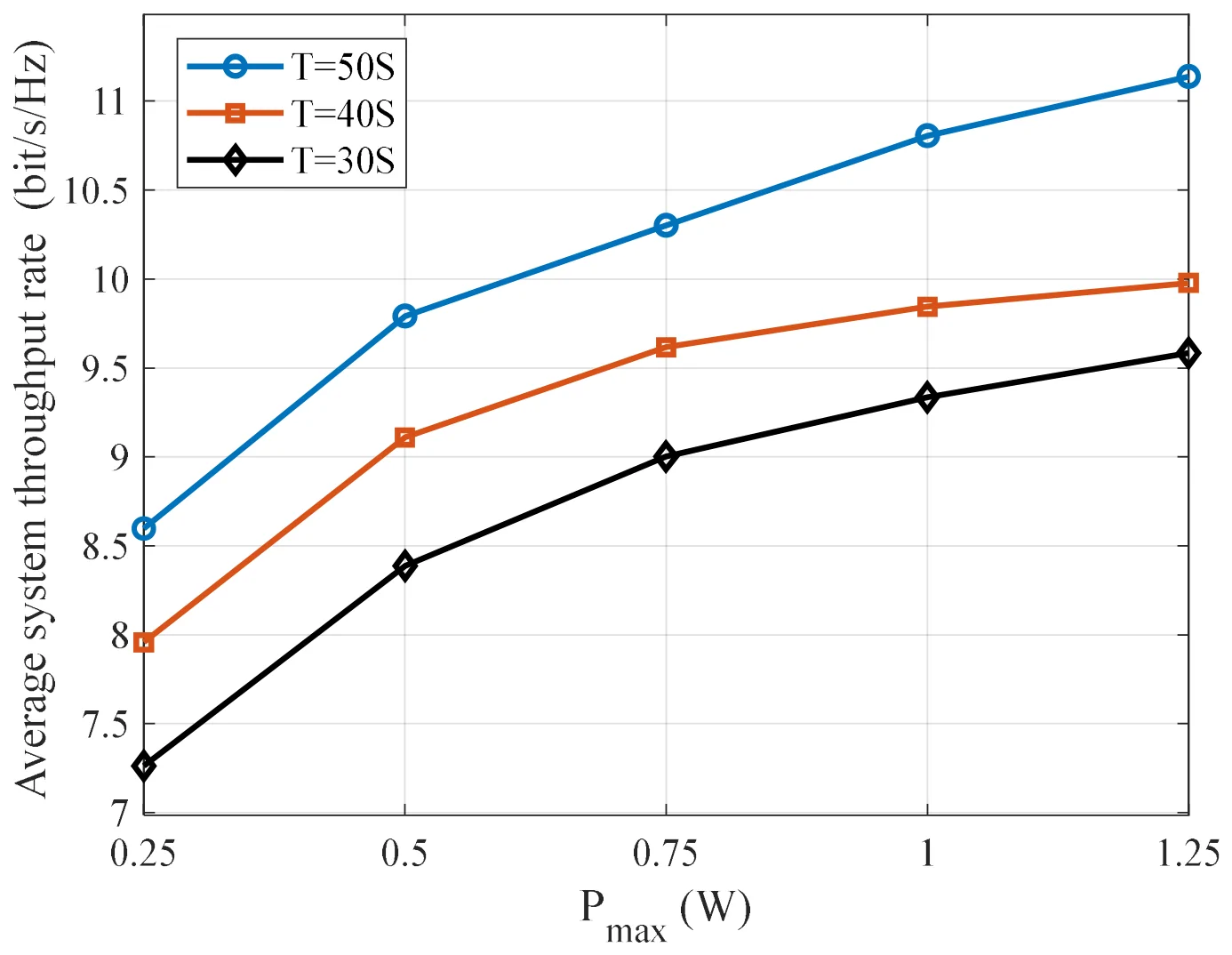
Average system communication rate versus the flight duration.

Figure 8 shows the variation in the average system communication rate with the transmit power under different flight durations. As the transmit power increases, the link quality improves and all curves rise accordingly. With a longer flight duration, the curves shift upward overall, indicating that the proposed joint trajectory and beamforming optimization can better exploit the additional flight time, allowing the UAV to stay at favorable locations for a longer period and thereby significantly improving the average communication rate.

**Figure 9:**
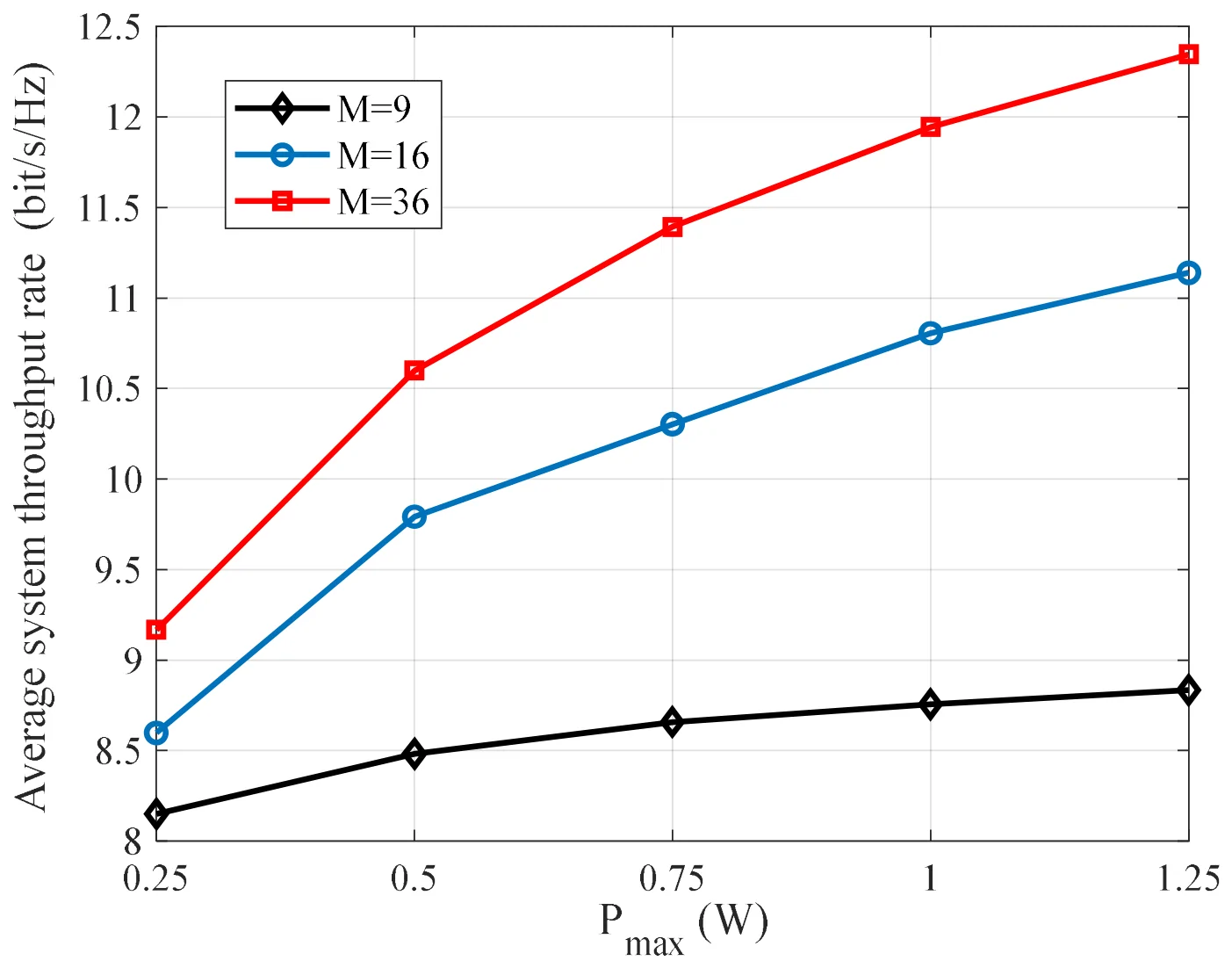
Average system communication rate versus the number of antennas.

Figure 9 presents the average system communication rate versus the transmit power under different antenna array sizes. As the transmit power increases, all curves increase monotonically, and a larger number of antennas leads to consistently higher curves. This is because a larger array provides higher array gain and stronger interference suppression capability. The proposed clustered RSMA beam design can effectively exploit these spatial degrees of freedom, thereby achieving higher spatial reuse efficiency.

**Figure 10:**
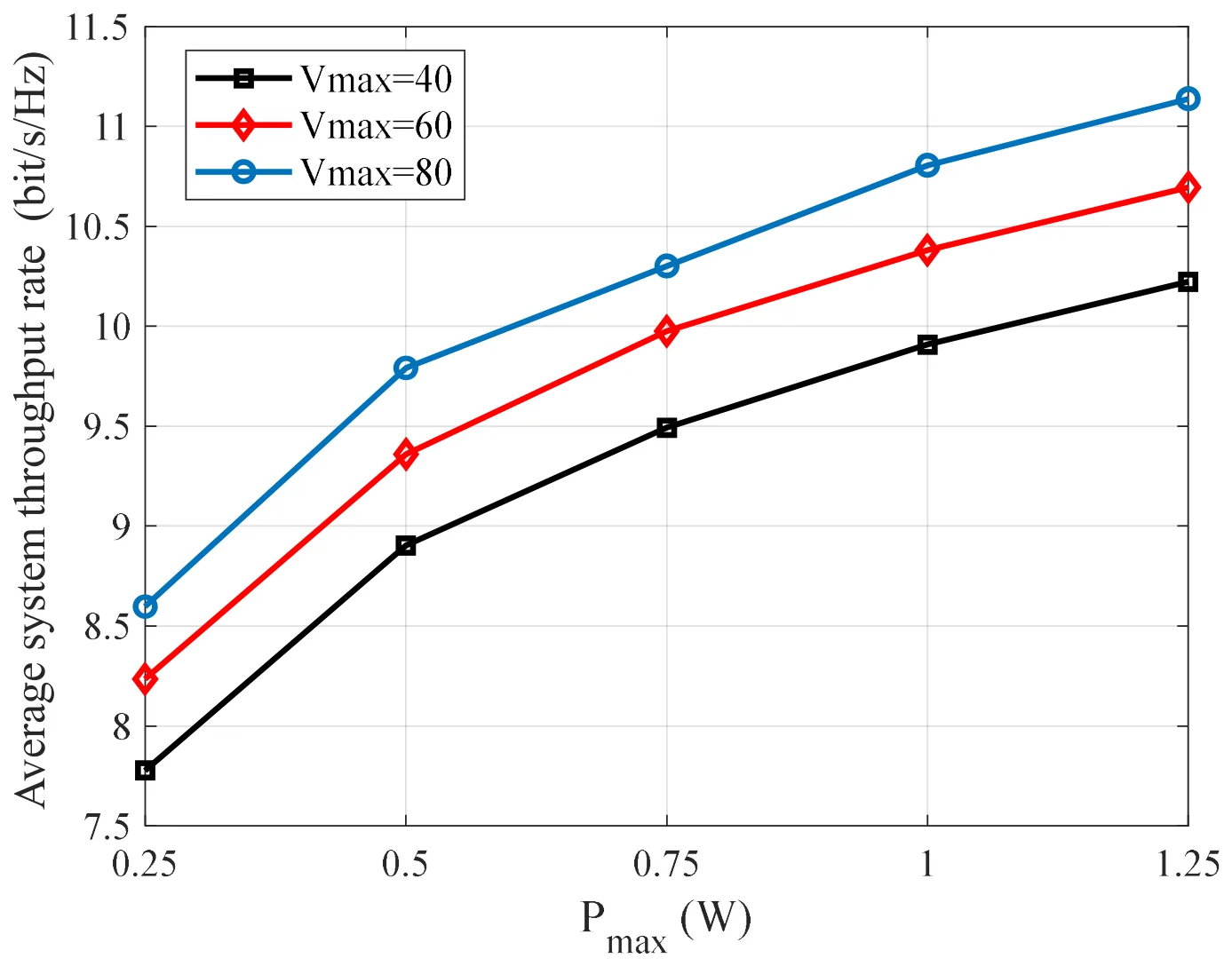
Average system communication rate versus the maximum UAV flight speeds.

Figure 10 illustrates the performance curves of the average system communication rate versus the transmit power under different maximum UAV flight speeds. As the transmit power increases, all curves rise, indicating that higher power is always beneficial for improving the link throughput. Moreover, a higher maximum flight speed results in higher curves, since stronger mobility enables the UAV to approach user clusters more quickly and reduces ineffective flying time. The proposed joint trajectory and beamforming optimization can effectively exploit this speed-related degree of freedom, thereby maintaining good overall communication performance even in high-speed platform scenarios.

## 5. Conclusions

This paper considers a UAV equipped with a UPA that simultaneously provides downlink communication and sensing services to ground users, and establishes a unified optimization framework for a clustered RSMA-UAV-ISAC system. The system performs spatial user clustering under a capacity–diameter constraint. Under constraints on user communication rates, transmit power, and sensing quality, an optimization problem is formulated to maximize the average downlink rate over a flight period. A block coordinate descent (BCD) approach is adopted to decompose the resulting non-convex problem into three subproblems: user scheduling, rate allocation and beamforming, and UAV trajectory design. These subproblems are solved using linear programming, semidefinite relaxation, and successive convex approximation, respectively. Through outer-loop alternating iterations, a converged feasible solution for cluster scheduling, common-rate allocation, common and private beamforming, and UAV trajectory is obtained. Simulation results demonstrate that the proposed algorithm can significantly improve the system communication rate while ensuring sensing quality. Nevertheless, the present work assumes quasi-static ground users and a free-space LoS channel model, and does not explicitly account for UAV propulsion energy. Future work will extend the proposed framework to mobile ground-user scenarios, 3GPP-based probabilistic LoS/NLoS channel models, and propulsion-energy-aware RSMA beamforming and UAV trajectory optimization. Multi-UAV cooperation and distributed joint communication and sensing will also be investigated.

## Figures and Tables

**Table 1:** Comparison with closely related RSMA-UAV-ISAC studies.

Work	Access Scheme	UAV Mobility	Sensing and Communication Mode	Optimized Variables	Main Limitation
Ref. [18]	RSMA	Static deployment	Simultaneous ISAC	UAV location and beamforming	The continuous UAV trajectory is not considered
Ref. [19]	Cooperative RSMA	Static UAV placement	Simultaneous ISAC	User association, UAV placement, beamforming, and rate allocation	A multi-UAV architecture is considered, while continuous trajectory design is not addressed
Ref. [23]	RSMA	Continuous trajectory	Time-division sensing and communication	Scheduling, power allocation, and UAV trajectory	Common and private beamforming vectors are not jointly optimized
This work	Clustered RSMA	Continuous trajectory	Simultaneous sensing and communication using the same waveform	Cluster scheduling, common-rate allocation, common and private beamforming, and UAV trajectory	A single-UAV scenario with simplified channel and sensing models is considered

**Table 2:** Simulation parameters.

Parameter	Value
UAV altitude: H	100 m
Flight period: T	50 s
Slot length: $\delta_{t}$	0.5 s
Carrier frequency: $f_{c}$	28 GHz
Antenna configuration:	UPA
Number of antennas: ($M_{x}$ $M_{y}$)	16
SIC detection threshold: ε	−90 dBm
Maximum transmit power: $P_{\max}$	0.5 W
Noise power spectral density: $\sigma^{2}$	−100 dBm
Path-loss constant: $\beta_{0}$	−61dB
Maximum UAV speed: $v_{\max}$	80 m/s
Minimum UAV speed: $v_{\min}$	0 m/s
Maximum number of users per cluster:	3
Minimum rate requirement: $R_{u}^{th}$	2 bit/s/Hz
Sensing threshold: $\Gamma_{l,k}^{th}$	0 dB

## Data Availability

Data is contained within the article.
